# Commentary: Mechanical Pain Thresholds and the Rubber Hand Illusion

**DOI:** 10.3389/fpsyg.2018.01715

**Published:** 2018-10-18

**Authors:** Matteo Martini

**Affiliations:** School of Psychology, University of East London, London, United Kingdom

**Keywords:** pain, body ownership, rubber hand illusion, virtual hand illusion, embodiment

Investigations into the relationship between bodily illusions and pain perception are representing a relatively modern trend in cognitive science. Recently, Bauer et al. (2018) published a work with the aim to determine if the vision of a potentially painful stimulus threatening the rubber hand can modify the mechanical pain threshold (MPT). They state that MPT remains relatively stable during the induction of the rubber hand illusion (RHI), yet it can be significantly decreased by the vision of an artificial threat to the RH. The purpose of the present commentary is to provide alternative explanations to Bauer's results, which have not been discussed in their article. This process would help promote additional reflection on this topic and hopefully foster further advances in this field.

The analgesic effects linked to the vision of the own body (“visual analgesia”) were initially described by Longo et al. (2009). Although there is not full consensus (Mohan et al., 2012; Torta et al., 2015), such effect has been consistently reported by many other studies (see Martini, 2016 for a review. For a neurophysiological explanation to this phenomenon please see the review written by Haggard et al., 2013). Discussing their main finding and supported by control experiments, Bauer and colleagues argue that their results stand in contrast to Longo's “visual analgesia” and they suggest that this apparent discrepancy might be due to the different material used in their experiments. I agree with the authors on the fact that the vision of a threatening stimulus can increase pain sensation (Arntz and Claassens, 2004; Höfle et al., 2012; Martini et al., 2013). While Bauer and colleagues threatened the rubber hand with a knife, clear threatening stimuli were not used in Longo's et al. study. However, there might be something else. In a recent virtual hand illusion (VHI) study, Nierula et al. (2017) set out to verify whether the distance between the real and the fake limb, typically present in RHI studies, could dampen visual analgesia. What they found was a significant decrease in heat pain thresholds when the virtual hand was far from the real hand compared to when they were perfectly co-located. So, visual analgesia is hindered if the real and the fake hand are not in the same place. The lack of analgesic effect due to the vision of “one' s own” body in Bauer's study could be then due to the distance (20 cm) between the real and the rubber hand. If this is true it might explain why, during the vision of the rubber hand being simply touched by the knife handle, there was no analgesic effect revealed by a higher MPT linked to the vision of “one' s own” body. Additionally, given the type of visual stimuli (knife point = threat vs. knife handle = no-threat) and the paradigm (RHI) used in their study, I think they should have discussed their findings also in the light of the latest findings on skin conductance response (SCR). Indeed, recent evidence point at an increase in the arousal response during the vision of stimuli approaching the owned rubber hand, regardless of the affective valence of the stimulus (Ma and Hommel, 2013; Johnson et al., 2016). So, the choice of a knife handle as a control stimulus could not be entirely appropriate.

In their third control condition Bauer and colleagues asked their participants to close their eyes before the measurement of MPT, so they did not see any stimuli approaching the RH. During this condition a modulation of proprioceptive drift was reported and a high level of ownership was found, but no pain modulation was documented. The authors thus state that “the induction of the *RHI alone* did not change the MPT values significantly” and that this would be in contrast with Martini et al. (2014). However, in the mentioned study all conditions envisaged constant visual feedback (i.e., no eyes closed) and the main finding was interpreted in favor of the transfer of the visual analgesia to virtual bodies, never mentioning a possible analgesic effect of the VHI “alone.” What precisely is this effect they refer to has to be clarified. Maybe the authors refer to another possible analgesic effect related to “disownership” of the real hand, which they state it did not take place. Unfortunately the phenomenon of disownership, likely overlapping the “loss of own hand” phenomenon (Longo et al., 2008), has not been directly measured by the authors. A future investigation specifically targeting the real contribution of the “disownership” phenomenon in pain studies with bodily illusion is therefore needed.

Another point worth discussing might be the type of pain chosen to measure the participants' pain threshold: the majority of studies about visual analgesia during RHI/VHI paradigms made use of thermal or electrical stimuli. In Bauer's experiment mechanical stimuli were chosen. The authors explain their preference stating that “MPT is assumed to be closer to clinical pain than thresholds measured with thermal stimuli,” but unfortunately no explanations nor any references were provided to support their assertion. Mechanical, electrical and heat pain threshold have been shown to have some level of independence and can react differently to different modulators (for ex. Tong et al., 2007; Okkerse et al., 2017). Furthermore, drawing on previous neurophysiological studies reporting a differential contribution of myelinated A-δ and unmyelienated C-fibers in different types of pain, Lötsch et al. (2016) have shown how electrical, thermal, and pinprick mechanical stimuli belong to three separate clusters of pain measures, and these stimuli seem to be processed differently in the brain (Murrell et al., 2007). Thus, the choice of the type of pain to gauge, as well as of other components of the experimental design (for ex. the choice of the control conditions), can make the difference in this type of experiments (Martini et al., 2015).

As a final point, given the high inter-subject variability and the complexity of the “embodiment” phenomenon (Longo et al., 2008), it might be always worthy reporting *qualitative* data too. What is a praxis for clinical research with patients could be extended to healthy participants as well, to boost interpretability of data and comparability among studies.

## Author contributions

The author confirms being the sole contributor of this work and has approved it for publication.

### Conflict of interest statement

The author declares that the research was conducted in the absence of any commercial or financial relationships that could be construed as a potential conflict of interest.
